# *Hydra*’s Lasting Partnership with Microbes: The Key for Escaping Senescence?

**DOI:** 10.3390/microorganisms10040774

**Published:** 2022-04-04

**Authors:** Jinru He, Thomas C. G. Bosch

**Affiliations:** Zoological Institute, Christian-Albrechts-University Kiel, 24118 Kiel, Germany; jhe@zoologie.uni-kiel.de

**Keywords:** *Hydra*, lamin, aging, non-senescence, stem cells, microbiota, metaorganism, cancer

## Abstract

Aging results from a complex interplay between genetic endowment and environmental exposures during lifetime. As our understanding of the aging process progresses, so does the need for experimental animal models that allow a mechanistic understanding of the genetic and environmental factors involved. One such well-studied animal model is the freshwater polyp *Hydra*. *Hydra* are remarkable because they are non-senescent. Much of this non-senescence can be ascribed to a tissue consisting of stem cells with continuous self-renewal capacity. Another important fact is that *Hydra*’s ectodermal epithelial surface is densely colonized by a stable multispecies bacterial community. The symbiotic partnership is driven by interactions among the microbiota and the host. Here, we review key advances over the last decade that are deepening our understanding of the genetic and environmental factors contributing to *Hydra*’s non-senescent lifestyle. We conclude that the microbiome prevents pathobiont invasion (colonization resistance) and stabilizes the patterning mechanisms, and that microbiome malfunction negatively affects *Hydra*’s continuous self-renewal capacity.

## 1. Introduction

Nature offers many different strategies of aging, from short-lived to long-lived species to “immortal” animals and plants. What underlies the aging process? In general, aging is a process that occurs as the regenerative potential of the organism diminishes. Both genetic and environmental factors contribute to the aging process. A landmark study [1] uncovered that the life span of nematodes can be doubled by a single genetic mutation in DAF-2, an insulin-like receptor upstream of a key regulator in aging and longevity, the transcription factor FOXO. FOXO is inactivated by signaling molecules such as insulin and insulin-like growth factor (IGF). People who have exceeded the age of 100 years remarkably often carry a sequence variation of the FOXO3A gene in their genome [2,3,4].

Moreover, a rapidly growing body of work shows that most if not all animals live in a close and interdependent relationship with a microbiome and, therefore, are regarded as metaorganisms [5]. The often substantial fitness effects of host–microbe interactions can drive the host and microbes to become deeply intertwined [6,7,8]. Dysbiosis or imbalances in these homeostatic host–microbiome interactions have repeatedly been linked to various diseases, including aging-related and end-of-life diseases [9]. The gut microbiota changes with aging with a decrease in diversity and core functions; and an enrichment of previously underrepresented bacteria that are potentially harmful [10]. Functional studies using microbial transplantation approaches in fish [11] and mouse models [12,13,14] point to a rejuvenation effect of the gut microbiota on aging-associated phenotypes. Although it is not yet clear to what extent aging drives changes in the gut microbiota or vice versa [9,15,16,17], there is consensus that the microbiome plays a fundamental role in aging and longevity [18]. This rapidly expanding realization of the critical importance of the microbiota to organismal health and aging implies the need of simple animal models in which one can experimentally manipulate both the host cells and the microbiome.

## 2. The Non-Senescent Metaorganism *Hydra*

One such well-studied animal model which allows an experimental and functional approach to understand host–microbe interactions and which at the same time is considered “immortal” or non-senescent” is the cnidarian polyp *Hydra*. Cnidarians represent a key transition in the evolution of animal complexity. A previous study based on demographic data uncovered that neither mortality nor fertility changes throughout *Hydra*’s lifespan, so that 5% of individuals remain alive after 1400 years under controlled lab conditions [19]. Lack of senescence in *Hydra* is due to the fact that all three stem cell lineages indefinitely maintain their self-renewal capacity, thus powering everlasting asexual growth [20,21]. Mortality is not changed in species like *Hydra vulgaris* AEP even after multiple rounds of sexual reproduction. Our experimental studies have shown that the transcription factor FOXO in *Hydra* modulates both stem cell proliferation and innate immunity, lending strong support to a role of FOXO as critical rate-of-aging regulator from *Hydra* to human [22,23].

As a metaorganism, *Hydra* is featured by a stable multispecies bacterial community which is densely colonizing the ectodermal epithelial surface [24]. Remarkably, since each *Hydra* species harbors a different set of bacteria [24,25], and since the total bacterial load is also strictly regulated [26,27], the *Hydra* host appears to impose specific selection pressure onto its microbiome. A rich repertoire of antimicrobial peptides produced in both ecto- and endodermal epithelial cells may directly regulate the microbiome [25,28]. The presence and composition of *Hydra*’s microbiota is critical for the tissue homeostasis and health of the polyps [29,30,31,32,33]. Since more than a decade we have been unravelling the intricate interactions between microbes and the non-senescent *Hydra*. *Hydra* has proven itself as an excellent model for researching host–microbe interactions and metaorganism functioning in vivo [32,34]. Here, we update the analysis with new findings and conclusions regarding the absence of senescence in *Hydra* and the evidence that symbiotic microbes play a contributory role in the maintenance of body function.

## 3. Non-Senescent *Hydra* Defies Common Denominators of Aging via Novel and Conserved Mechanisms

Many studies have shown that alteration of the components of the nuclear lamina is associated with aging, both in vivo and in vitro. When analysing the role of a single lamin protein in *Hydra*, we have discovered to our surprise that the disturbance of Lamin expression and localization does not change stem cell proliferation [35]. Gain- and loss-of-function experiments uncovered that while Lamin is indispensable for *Hydra*, the stem cells tolerate overexpression, downregulation and mislocalization of Lamin and the resulted modification of nuclear envelope structure. We conclude that non-senescence and indefinite self-renewal capacity of stem cells is coupled with a relatively simple nuclear envelope architecture with an extraordinary robustness.

Looking at the DNA repair mechanisms, *Hydra* is not much different from other animals. *Hydra* genes encoding DNA repair pathways matched confidently with their vertebrate orthologues, indicating conservation at the level of sequence, structure, and function [36]. When genomic DNA of UV-exposed *Hydra* was checked for repair of cyclobutane pyrimidine dimers (CPDs) [36], most of the CPDs were found to be repaired by 72 h, confirming that *Hydra* repairs damaged DNA. A high expression level of DNA repair protein XPF in the multipotent gamete-producing interstitial stem cells and the presence of the base excision repair (BER) and nucleotide excision repair (NER) pathway point to an effective toolbox for DNA repair and maintenance of genome integrity. Although DNA damage plays a causal role in aging, the finding of a conserved toolbox of DNA repair genes does not necessarily explain the ability to avoid cellular senescence. The same applies to another mode of DNA protection in the form of the enzyme telomerase. Telomeres play a central role in aging by adjusting the cellular response to stress and growth stimulation caused by previous cell divisions and DNA damage. Molecular damage in the form of nuclear mutations may accumulate even in the most long-lived organisms over time. When characterizing the telomerase of *H. vulgaris*, Skvortsov et al. (pers. commun.) discovered that the expression level of telomerase catalytic subunit (hyTERT) mRNA corresponded to regions with high proliferative potential and did not change during budding or regeneration. The results were confirmed by measuring telomerase activity (Dmitry Skvortsov and Feodor M Eroshkin, pers comm). However, whether *Hydra* cells use a continuously functioning telomerase to counteract telomere shortening and to escape cellular aging, requires further research to confirm.

## 4. Life at the Surface of *Hydra*´s Ectodermal Epithelium

Ectodermal epithelial cells are covered by an extracellular cuticle (“glycocalyx”) with a complex layered structure consisting of proteins and glycosaminoglycans. The glycocalyx has conserved mucus-like properties and provides the habitat for the symbiotic bacterial community [32,37]. In similarity to the intestinal mucus in mammals, the constant renewal of this mucus-like layer most likely allows *Hydra*’s rapid adjustments to a constantly changing environment [32]. *Hydra*´s microbiota located on the surface of the ectodermal epithelium is characterised by members of the bacterial genera *Curvibacter*, *Pseudomonas*, *Acinetobacter* and *Duganella*. These microbes grow by utilizing nutrients provided by their host [38]. The *Hydra* polyp shapes the specific microbiome by means of the innate immune system and a rich repertoire of antimicrobial peptides which are secreted by epithelial cells [25,28] as well as ectodermal neurons [39]. The findings reveal that in *Hydra* epithelia and components of the innate immune system play an active role in selecting the inhabitant microbiota via a complex genetic network. Microbial metabolites not only stimulate *Hydra*’s immune response but also influence behavior [39,40].

## 5. Longevity Factor FOXO Controls Microbiome Resilience in *Hydra*

The transcription factor FOXO, which regulates genes involved in growth and differentiation has been consistently associated with human aging and longevity [4]. In *Hydra*, the single FoxO gene is strongly expressed in all stem cell lineages [22]. In genetic experiments, we were able to show that increased FOXO protein synthesis leads to increased cell division and causes even differentiated *Hydra* cells to take on a stem cell character [22].

Surprisingly, a deficiency in FOXO signaling leads not only to malfunctions in cell cycle progression but also to dysregulation of multiple families of genes encoding antimicrobial peptides (AMPs) [41]. Most genes encoding epithelially expressed AMP families, including Hydramacin, Kazal, and Arminin, are downregulated in response to FOXO deficiency, suggesting a mainly activating function of FOXO signaling on AMP expression and innate immunity. FOXO loss-of function polyps were more susceptible to colonization by foreign bacteria and impaired in maintaining bacterial composition resembling the native microbiome. Thus, a FOXO-induced decrease in AMP expression is coupled to shifted microbial colonization and highlights the inhibitory function of AMPs against non-commensal bacteria. Consequently, especially during the process of colonization, downregulation of FOXO compromises the resilience of the microbiome. Downstream of FOXO, secretion of numerous AMP families appears to provide a highly selective milieu shaping the species-specific microbial composition [28]. Taken together, non-senescent *Hydra* research supports the view that FOXO establishes a direct link between the division and regeneration capacity of stem cells and the maintenance of a stable microbiome essential for development and health, adding an important dimension to aging biology that has been poorly understood.

## 6. The Microbiome Modulates *Hydra*´s Tissue Homeostasis

The microbiota has a profound impact on both form and functioning of *Hydra*´s epithelial body architecture. Both epithelial layers not only self-renew but also independently modulate development and morphogenesis. Classic experiments using chimeric strains [42] showed that the ectoderm regulates body morphology, while the endoderm controls body size [42]. This decades-old observation takes on a whole new meaning, because recent observations show that body size in *Hydra* is determined not only by internal regulatory processes, but also by environmental factors including temperature and the microbiome [43,44]. The observation that germ-free polyps grow larger in size than conventionalized animals [44] suggests the potential importance of the microbiome in size determination [45].

Recent studies uncovered that in *Hydra* WNT/β-catenin signaling is stabilized by microbial colonization. Regeneration and transplantation experiments performed as early as 1909 assigned the organizer function to the tip of the *Hydra* head [46], and the formation of the head organizer involves the canonical Wnt pathway [47,48,49]. Treatment with alsterpaullone (ALP), a GSK3-3β inhibitor, results in increased activity of the head organizer system in the body column [48]. Intriguingly, germ-free *Hydra* polyps are significantly more sensitive to ALP than control polyps associated with microbes [50]. Germ-free animals grow four times more ectopic tentacles after ALP treatment compared to control animals. It thus seems that the presence of the microbiota affects maintenance of the proliferation zone and terminal differentiation processes. Differential gene expression analysis on the impact of bacterial colonization identified four *Hydra* genes that were upregulated by bacterial colonizers and showed spatially and temporally regulated expression patterns [50]. Further screening revealed a small, secreted protein, named Eco1a, being upregulated in the response to both bacterial colonizers and low temperatures. Transgenic studies showed that this novel microbe-dependent effector gene is indeed involved in the regulation of pattern formation and has an antagonistic function to Wnt signaling in *Hydra* [50]. Recent observations on long-term germ-free animals provide direct support for the view that *Hydra*’s developmental programs are dependent on the microbiota [44]. This dependence involves gene regulatory networks and signaling pathways that are highly conserved in the animal kingdom. It thus seems that non-senescent *Hydra* not only can provide important insights into general principles of biological pattern formation but also uncover the hidden impact of the microbiome for at least some conserved developmental pathways

## 7. Microbiome Malfunction Drives Cancer Development in *Hydra*

Cancer is often defined as a disease of aging [51]. The incidence of most cancers increases dramatically as we age and is primarily caused by the disruption of normal cellular functions through genetic, epigenetic, and paracrine changes. The recognition that microbes can not only be potential causes of cancer but also play a crucial role in the efficacy of anticancer therapies [52,53,54] begs the question: does the normal microbiome play a role in the development of cancer? Cancer arose after the transition to multicellularity early in the evolution of life. Several years ago, Domazet-Lošo and colleagues reported tumor naturally occurred in *Hydra*, and demonstrated that in principle all multicellular animals can form tumors [55]. Using the experimental accessibility of the *Hydra* metaorganism, recent research has demonstrated that following microbial composition shifts due to changes of environmental factors, the tissue can be colonized by foreign bacteria. In our study [33], these are often spirochaetes. Interestingly, only in the co-occurrence with a symbiotic bacterium from the genus *Pseudomonas*, spirochaetes become a virulent and tumor-forming agent in *Hydra*´s epithelium. When spirochaetes encounter *Pseudomonas*, which itself is part of the normal composition of the *Hydra* microbiome, the two bacterial species immediately change their transcriptional profile and activate factors that have a pathogenic effect for the host organism. Due to these changes, the microbiota composition becomes drastically altered followed by structural changes in the *Hydra* epithelial cells and ultimately leading to tumor formation. Thus, it is probably not a single pathogenic intruder, but the malfunction of the microbiome as a protective barrier for the body as a whole that can promote the development of cancer in *Hydra*. Under stable environmental conditions microbial colonization seems to protect the non-senescent organism against harmful invaders, potentially even against carcinogenic influences. How these interactions occur at the molecular level, and which specific biochemical mechanisms are involved in this form of cancer development, are the subject of currently ongoing investigations.

## 8. Concluding Remarks and Future Perspectives

Aging is a universal phenomenon, which occurs in different degrees in all multicellular species. Comparative approaches based on the diversity of aging strategies found in nature, therefore, are not only highly useful to better understand the mechanisms that regulate aging. They also help to uncover unifying principles on the mechanisms and evolution of the aging phenomenon. Our observations in non-senescent *Hydra* allow three important general conclusions. First, since aging is a fundamentally multi-organismal process, there is a need to consider the multi-organismic and holobiotic nature of an organism surrounded by its microbial counterparts when thinking about longevity. Second, the microbial environment matters in the context of senescence and contributes to complex processes such as aging. Third, well-established hub regulators including WNT/β-catenin signaling and FOXO present a direct link between age-related processes and microbial colonization. Offering a new and evolutionary informed perspective into the study of the role of host–microbe interactions in aging, we argue here that decoding the mechanisms of how non-senescent *Hydra* communicates with its microbiome can provide a general understanding of the tightly linked interactions between host environment, nutrient dependency of host-associated microbes, microbial metabolism, microbe–microbe interactions and host immunity.
